# Psychometric Validation of the Dutch Version of the Promotive and Prohibitive Voice Scale

**DOI:** 10.3389/fpsyg.2021.722238

**Published:** 2021-11-25

**Authors:** Roy B. L. Sijbom, Jessie Koen

**Affiliations:** Department of Work and Organizational Psychology, University of Amsterdam, Amsterdam, Netherlands

**Keywords:** validation, employee voice, measurement invariance, reliability, nomological network

## Abstract

The aim of this three-study report was to validate the Dutch version of the promotive and prohibitive voice scale and to further embed the constructs of promotive and prohibitive voice within their nomological network. Promotive voice refers to the expression of suggestions for improving work practices, whereas prohibitive voice refers to the expression of concerns about practices and behaviors that are detrimental. In Study 1 (*N* = 121), confirmatory factor analyses (CFAs) provided evidence for the two-factor structure, which was replicated in the other two studies. In Study 2 (*N_*T*1_* = 209/*N_*T*2_* = 107), we investigated the convergent and discriminant validity of the promotive and prohibitive voice scale, and tested measurement invariance across gender and time. Results provided validity evidence, partial scalar invariance for gender, and scalar invariance across time. In Study 3 (*N* = 149), we expanded the nomological network of the promotive and prohibitive voice scales through their relationship with personal initiative, approach temperament, and risk propensity. Taken together, our results provide strong evidence for the validity of the Dutch version of the promotive and prohibitive voice scale.

## Introduction

Employee voice –the expression of constructive opinions, concerns, or ideas about work-related issues– is crucial to the functioning of organizations (Van Dyne et al., 2003; Morrison, 2011). For example, employee voice behavior contributes to the innovative capacity of organizations, learning within teams, and improving efficiency (Morrison, 2014). Voice behavior can also prevent problems and malfeasance within organizations, such as medical malpractice. Given the positive outcomes of voice behavior for organizations, much research has been conducted in recent years on factors and conditions that can encourage voice behavior (e.g., Liu et al., 2015; Kakkar et al., 2016).

Although the definition of employee voice entails constructive suggestions, its manifestation may differ. For example, employees can formulate ideas for improving procedures, or can speak up when a colleague undermines team performance. To account for these different manifestations, scholars have differentiated the voice construct based on its content domain: promotive (or suggestion-focused) voice and prohibitive (or problem-focused) voice (Morrison, 2011; Liang et al., 2012). Promotive voice refers to the expression of suggestions for improving current practices, whereas prohibitive voice refers to the expression of concerns about practices and behaviors that might have detrimental effects. Distinguishing between these two manifestations of employee voice is important because suggestions for improvement are just as important for organizations as identification and reporting of errors and problems. In fact, both are required for a healthy functioning of organizations (Argyris, 1977).

To adequately differentiate between these different manifestations of employee voice, Liang et al. (2012) developed and validated a new employee voice scale. This new scale measures promotive and prohibitive forms of voice. Since its introduction, this voice scale has been used as the new standard for studying employee voice and has advanced the field of voice research. That is, a cumulating body of empirical evidence indicates that differentiating between promotive and prohibitive voice is of great theoretical value. To illustrate, a meta-analysis by Chamberlin et al. (2017) has shown that different antecedents, such as core self-evaluations and ethical leadership, are differentially related to promotive and prohibitive voice. Likewise, promotive and prohibitive voice are differentially related to outcomes such as job performance (Chamberlin et al., 2017). These insights are also of great practical value, as they allow organizations to stimulate specific types of voice behavior. Thus, given the theoretical and practical value of distinguishing between promotive and prohibitive voice, it is essential that validated versions of the promotive/prohibitive voice scale are available in different languages. Such translated versions would help to expand cross-cultural comparisons and, hence, to further advance research on employee voice. With this study, we aim to provide a validated measure of promotive and prohibitive voice in Dutch.

Although translated versions of the promotive/prohibitive voice scale developed by Liang et al. (2012) do exist - for example, the scale was used in a sample from a Chinese bank (Huang et al., 2018), in a sample of Norwegian white-collar workers (Svendsen et al., 2018), and in a German sample (Cheng et al., 2020) -, the psychometric properties of the scale have remained understudied and, hence, unclear. In fact, no validated translated versions of the promotive and prohibitive voice scale have been published, the invariance of the structure of the scales is generally assumed but hardly tested (for an exception, see Svendsen et al., 2018), and there is little information available on the construct validity and the nomological network of the scale. This is problematic for multiple reasons. First, without evidence on the construct validity (i.e., convergent validity, discriminant validity, and nomological network) of the translated promotive and prohibitive voice scale, the interpretations of empirical results on promotive and prohibitive voice remain obscure. Second, without having measurement invariance tested, we cannot assume that a construct has the same meaning across groups or across repeated measurements (Vandenberg and Lance, 2000; Putnick and Bornstein, 2016). It is essential that participants across all groups and across all time points interpret the scale items in the same way. Only then, it is possible to make comparisons between groups (e.g., gender) regarding their voice behavior and to draw conclusions from longitudinal designs. Third, the lack of validated scales in other languages limits cross-cultural comparisons. While the promotive and prohibitive voice scales are used in different countries and languages, validated translations of the scale need to be available in order to conduct meaningful and methodologically sound voice research across the world.

With this multi study report, we aim to provide evidence for the psychometric properties of the Dutch version to measure promotive and prohibitive voice. In order to do so, we will (a) investigate convergent and discriminant validity, (b) test measurement invariance across groups and across time, and (c) aim to advance the divergent nomological networks of promotive and prohibitive voice. The resulting psychometrically sound measure has the potential to expand cross-cultural comparisons and to advance voice research in general and in the Dutch-speaking scientific community specifically. In the following, we will discuss our expectations regarding convergent and discriminant validity, measurement invariance, and the nomological network.

### Convergent Validity and Discriminant Validity

Convergent validity is demonstrated when an instrument shows positive and rather high associations with instruments that are intended to study theoretically similar concepts (Campbell and Fiske, 1959). To examine the convergent validity of the Dutch version of the promotive and prohibitive voice scale, we will assess how this scale correlates with two other employee voice measures that are frequently used: the employee voice scale from LePine and Van Dyne (1998) and the employee voice scale from Farh et al. (2007). Like the promotive and prohibitive voice scale, these employee voice scales also aim to measure proactive behavior with the intent to improve the workplace. As such, we expect that our promotive and prohibitive voice scale will be positively related to these two alternative voice scales. Yet, the scales of LePine and Van Dyne (1998) and Farh et al. (2007) focus on expressions of opportunities to enhance organizational functioning by doing new things (i.e., promotive voice), rather than on expressions intended to benefit the organization by preventing negative consequences (i.e., prohibitive voice). Accordingly, we expect that promotive voice will be more strongly related to these two alternative measures of employee voice than prohibitive voice.

Discriminant validity reflects the extent to which a measure does not relate to constructs from which it should theoretically differ, indicated by moderate to low correlations (Cronbach and Meehl, 1955). To examine the discriminant validity of the Dutch version of the promotive and prohibitive voice scale, we will assess how the scale correlates with two constructs: knowledge sharing and employee silence. The first construct, knowledge sharing, is defined as “the act of making knowledge available to others within the organization” (Ipe, 2003, p. 341). While knowledge sharing and voice both involve the exchange of information, they substantially differ in two important ways. First, knowledge sharing can be seen as an individual learning strategy (Lu et al., 2012), whereas voice is focused on improving the unit and organization as a whole. Second, knowledge sharing is considered necessary to execute formal tasks, whereas voice is considered a proactive behavior that is not part of formal working procedures. Accordingly, we expect that promotive and prohibitive voice will show moderate to low correlations with knowledge sharing. The second construct, employee silence, refers to the conscious withholding of potentially important information, suggestions, or concerns, from those who might be able to act on that information (Van Dyne et al., 2003). Although voice and silence might seem like opposites on the same continuum, research clearly illustrates that they are orthogonal (Sherf et al., 2021). As such, we expect that promotive and prohibitive voice will show moderate to low correlations with employee silence.

### Measurement Invariance

Although many studies assume that different groups have the same understanding of the promotive and prohibitive voice construct and that the structure of the scales remains the same in different groups and over time, this assumption has almost never been appropriately tested (for an exception, see Svendsen et al., 2018). This is unfortunate, because without such evidence we cannot be sure that we are measuring the same construct across groups and over time (Vandenberg and Lance, 2000). Put differently, before any justified comparisons between groups or over time can be made, measurement invariance, i.e., “the psychometric equivalence of a construct across groups or across time” (Putnick and Bornstein, 2016, p. 71), should be demonstrated. Therefore, we will investigate the measurement invariance of the Dutch version of the promotive and prohibitive voice scales across gender and time.

### Nomological Network

Finally, to further establish the construct validity of the Dutch version of the promotive and prohibitive voice scales, we will investigate and extend its nomological network with three dispositional antecedents: personal initiative, approach temperament and risk propensity. The first antecedent, personal initiative, refers to a behavioral style characterized by taking a proactive, self-starting approach to work, going beyond formal job requirements, and demonstrating persistence in overcoming barriers (Frese et al., 1997; Frese and Fay, 2001). Meta-analytical evidence shows that personal initiative is the most important individual dispositional antecedent of voice (Chamberlin et al., 2017), although no differentiation was made between different types of voice. Given that both promotive and prohibitive voice hinge on proactivity and the willingness to go beyond role requirements and allocate effort on behalf of the organization, we expect personal initiative to be positively associated with both promotive and prohibitive voice.

The second antecedent, approach temperament, is defined as “a general neurobiological sensitivity to positive (i.e., reward) stimuli (present or imagined) that is accompanied by perceptual vigilance for, an affective reactivity to, and a behavioral predisposition toward such stimuli” (Elliot and Thrash, 2010, p. 866). The approach and change-related aspects reflected in approach temperament bear similarities with promotive voice rather than with prohibitive voice: promotive voice is focused on improving and aiming for positive outcomes by signaling and expressing opportunities for doing new things. As such, we expect approach temperament to be positively related to promotive voice in particular.

The third antecedent, risk propensity, refers to people’s willingness to take risk (Sitkin and Weingart, 1995). Research shows that people’s assessment of perceived risk is one of the most important underlying factors in expressing prohibitive voice behavior (Wei et al., 2015). For example, because prohibitive voice is focused on problems or sensitive topics, it might jeopardize individual relationships. Given these involved risks, the decision to voice involves a calculated decision process in which individuals assess the likelihood that they will be successful as well as the likely consequences of their action, such as whether the risks of voicing outweigh the benefits (Morrison and Phelps, 1999). The core argument is that individuals are more likely to engage in voice as their judgments of safety increase (Morrison, 2014). People’s risk propensity can be expected to lead to higher levels of perceived safety and therefore also in more prohibitive voicing. Accordingly, we expect risk propensity to be positively associated with prohibitive voice in particular.

### Overview of Current Studies

The aim of the present study was to validate and test the psychometric properties of the Dutch version of the promotive and prohibitive voice scale (based on Liang et al., 2012). To do so, we conducted three studies. In Study 1, we conducted a confirmatory factor analysis (CFA) to verify the two-factor structure of the Dutch voice scale, and tested the internal consistency of the promotive and prohibitive voice scale to ensure it exceeded the recommended cutoff of 0.70 (Hinkin, 1998). In Study 2, we investigated the convergent and discriminant validity of the promotive and prohibitive voice scale and tested measurement invariance across gender and time. In Study 3, we advanced the nomological network by investigating the relationships of both voice scales with personal initiative, approach temperament, and risk propensity.

## Study 1

### Method

#### Sample and Procedure

A convenience-sampling method was used to collect the data. Data were collected by a trained master student as part of a research project at their University, under the first author’s supervision. Participants were invited by email or via professional network contacts (e.g., LinkedIn) to participate in the study. This sample consisted of Dutch employees (*N* = 121) who were employed at several consultancy firms in the Netherlands. Their age ranged from 21 to 64 years, with a mean age of 36.7 years (*SD* = 12.38; based on *N* = 105). Of the 121 employees, 81 (66.9%) were female, 38 (31.4%) were male, and two (1.7%) did not answer this question. Fifty-six percent of the employees had a permanent contract, 38% a temporary contract, and 6% did not answer this question. Mean organizational tenure in the current organization was 6.8 years (*SD* = 8.7). The questionnaire was administered online. The study was approved by the University’s Ethics Review Board (2017-WOP-7995).

#### Measures

##### Promotive and Prohibitive Voice

Promotive and prohibitive voice was assessed with a translated Dutch version of the 10-item scale (see Appendix Table A1) developed by Liang et al. (2012). Five items measured promotive voice (“I proactively develop and make new suggestions for issues that may influence the unit/organization”) and five items measured prohibitive voice (“I advise other colleagues against undesirable behaviors that would hamper job performance”). Items were rated on 5-point Likert scale ranging from 1 (*strongly disagree*) to 5 (*strongly agree*). We translated the original items (Liang et al., 2012) to Dutch using a translation-back-translation procedure (Brislin, 1986).

#### Data Analysis

Confirmatory factor analyses (CFAs) using AMOS 25.0 (Arbuckle, 2017) were conducted to validate the factor structure of the translated items. We compared a two-factor model with a one-factor model, and assumed the former would fit the data best. To evaluate the model fit we considered the χ^2^-value divided by the degrees of freedom (χ^2^/df), the Root Mean Square Error of Approximation (RMSEA), the Standardized Root Mean Residual (SRMR), the Tucker–Lewis Index (TLI), and the Comparative Fit Index (CFI). Typically, χ^2^/df ratio < 2, RMSEA < 0.08, SRMR < 0.09, and TLI and CFI > 0.90 indicate an acceptable fit (Hu and Bentler, 1999). To test the two-factor model, the two sets of five items were used as indicators for the promotive and prohibitive voice scales, respectively. The promotive and prohibitive voice scales were allowed to correlate at the latent level.

### Results and Discussion

The mean for promotive voice was 3.85 (*SD* = 0.63; range = 2.20–5.00) and the mean for prohibitive voice was 3.45 (*SD* = 0.59; range = 2.00–4.80). Skewness and kurtosis values for the promotive voice items ranged from (−0.65 to −0.30) and (−0.20 to 0.90), respectively. For the prohibitive voice items, skewness and kurtosis values ranged from (−0.60 to −0.08) and (−1.04 to 0.22), respectively. These results suggest that the promotive and prohibitive voice items conform to the assumptions of correlation-based statistics for this sample (cf. Cohen et al., 2003). Cronbach’s alpha values were 0.86 for promotive voice and 0.74 for prohibitive voice, indicating acceptable to good reliability (Tavakol and Dennick, 2011). The intercorrelation between promotive and prohibitive voice was significant (*r* = 0.48, *p* < 0.001) and comparable to results found in previous studies (cf. Liang et al., 2012; Kakkar et al., 2016).

Table 1 presents the fit indices of the tested factor-structure models. The two-factor model showed an acceptable fit and was significantly superior to a one-factor model [Δχ^2^ (1) = 54.17, *p* < 0.001]. All standardized factor loadings within the two-factor model were significant and ranged from 0.74 to 0.76 for the promotive voice factor and from 0.50 to 0.73 for the prohibitive voice factor (see Table 2). In addition, we calculated the values of average variance extracted (AVE). The AVE value for promotive voice (0.56) was above the suggested cutoff of 0.50 (Fornell and Larcker, 1981). The AVE value for prohibitive voice (0.37) was below the suggested cutoff of 0.50. Yet, given that the composite reliability (CR) for both promotive voice and prohibitive voice were above 0.70 (0.86 and 0.74, respectively), convergent validity can still be regarded as adequate (Fornell and Larcker, 1981). Together, these results show that the Dutch version of the promotive and prohibitive voice scales are internally consistent and provided initial evidence for the instrument’s two-factor structure.

**TABLE 1:** Fit statistics for the measurement models.

Models	χ^2^ (df)	χ2/df	CFI	TLI	RMSEA (90% CI)	SRMR	Model comparison	Δχ2 (df)	Δ CFI	Δ TLI	Δ RMSEA
**Study 1**											
M1: 2 latent factors	66.80* (34)	1.965	0.922	0.897	0.090 (0.057–0.121)	0.065					
M2: 1 latent factor	120.97** (35)	3.456	0.795	0.737	0.143 (0.116–0.171)	0.100	M1	54.17** (1)	0.127	0.160	0.053
**Study 2**											
M1: 2 latent factors	88.30** (34)	2.597	0.954	0.939	0.088 (0.065–0.110)	0.060					
M2: 1 latent factor	339.82** (35)	9.709	0.742	0.668	0.205 (0.185–0.225)	0.119	M1	251.52** (1)	0.212	0.271	0.117
**Study 3**											
M1: 2 latent factors	69.28* (34)	2.038	0.951	0.935	0.084 (0.055–0.112)	0.051					
M2: 1 latent factor	176.92** (35)	5.055	0.801	0.745	0.166 (0.142–0.190)	0.104	M1	107.64** (1)	0.150	0.190	0.082
**Combined sample^a^**											
M1: 2 latent factors	93.00** (34)	2.735	0.975	0.966	0.060 (0.046–0.075)	0.042					
M2: 1 latent factor	504.72** (35)	14.420	0.797	0.740	0.168 (0.155–0.181)	0.106	M1	411.72** (1)	0.178	0.226	0.108

*p < 0.05

**p < 0.001.

*^a^This consists of the samples of Study 1, Study 2 (time 1), and Study 3.*

**TABLE 2:** Estimated standardized factor loadings of the Dutch promotive and prohibitive voice scales in Study 1 (*N* = 121), Study 2 (*N*_*T1*_ = 209/*N*_*T2*_ = 107), Study 3 (*N* = 149), and combined sample (*N* = 479).

		Standardized factor loadings Study 1	Standardized factor loadings Study 2	Standardized factor loadings Study 3	Standardized factor loadings combined sample
**Promotive voice**					
(1)	I proactively develop and make suggestions for issues that may influence the unit.	0.75	0.71/0.76	0.78	0.75
(2)	I proactively suggest new projects which are beneficial to the work unit.	0.74	0.75/0.67	0.72	0.74
(3)	I raise suggestions to improve the unit’s working procedure.	0.76	0.82/0.88	0.84	0.81
(4)	I proactively voice out constructive suggestions that help the unit reach its goals.	0.74	0.86/0.90	0.81	0.82
(5)	I make constructive suggestions to improve the unit’s operation.	0.76	0.84/0.78	0.81	0.82
**Prohibitive voice**					
(6)	I advise other colleagues against undesirable behaviors that would hamper job performance.	0.52	0.69/0.74	0.65	0.68
(7)	I speak up honestly with problems that might cause serious loss to the work unit, even when/though dissenting opinions exist.	0.57	0.72/0.72	0.73	0.70
(8)	I dare to voice out opinions on things that might affect efficiency in the work unit, even if that would embarrass others.	0.68	0.81/0.79	0.79	0.75
(9)	I dare to point out problems when they appear in the unit, even if that would hamper relationships with other colleagues.	0.73	0.89/0.83	0.67	0.79
(10)	I proactively report coordination problems in the workplace to the management.	0.50	0.64/0.63	0.66	0.64

## Study 2

In Study 2, we aimed to provide further evidence for the psychometric properties of the Dutch version of the promotive and prohibitive voice scales. For this purpose, we (1) again assessed the validity of the two-factor structure, (2) tested the convergent and discriminant validity of the scales, and (3) investigated measurement invariance across gender and across time.

### Method

#### Sample and Procedure

Data were collected by trained bachelor students as part of a research project at their University, under the first author’s supervision. Students first collected email addresses of employees from Dutch organizations in several industries. The first author then sent an online survey to these employees (Time 1), and a follow-up survey 3 months later (Time 2). At the start of each survey, a cover letter explained the nature of the research and assured participating individuals of anonymity and confidentiality. Participants digitally signed an informed consent. The study was approved by the University’s Ethics Review Board (2020-WOP-12208).

At Time 1, 229 out of 322 approached employees finished the questionnaire. From 20 employees data on relevant study variables was missing, leaving a final sample of 209 for the analyses (response rate T1: 64.9%). Seventy-five employees were male (35.9%) and 134 were female (64.1%). Their age ranged from 18 to 66 years, with a mean age of 36.0 years (*SD* = 14.79). One hundred forty-five (69.4%) of the employees had a permanent contract, and 64 employees (30.6%) had a temporary contract. Mean tenure was 16.5 years (*SD* = 13.5). The main sectors represented in the sample commercial services (17.2%), healthcare (14.4%), financial services (8.6%), education (7.7%), retail (7.2%), government (6.2%), and catering and recreation (6.2%). At Time 2, a total of 128 out of 229 employees started the follow-up questionnaire, of which 111 provided data on relevant study variables (response rate T2: 48.5%). After matching employees who had completed both questionnaires, the sample consisted of 107 participants (response rate: 33.2%).

#### Measures

Unless indicated otherwise, all measures were assessed with 5-point Likert scales ranging from 1 (*totally disagree*) to 5 (*totally agree*). All measures were assessed at Time 1. To test measurement invariance across time, we again assessed promotive and prohibitive voice at Time 2.

##### Promotive and Prohibitive Voice

Promotive and prohibitive voice were assessed with the 10-item Dutch version of the voice scale as described in Study 1.

##### Knowledge Sharing

We assessed knowledge sharing with a 5-item scale, with four items derived from the Knowledge Sharing scale of Nerstad et al. (2018), e.g., “I share information I have with my colleagues,” and one item derived from Reinholt et al. (2011): “I am willing to share my knowledge with my team members.”

##### Employee Silence

We used the 5-item scale from Tangirala and Ramanujam (2008) to measure employee silence. An example item is “I chose to remain silent when I had concerns about problems in our workgroup”.

##### Employee Voice

Employee voice was measured with two alternative voice scales, similar to Liang et al. (2012). The first employee voice scale from LePine and Van Dyne (1998) consisted of four items, e.g., “I speak up and encourage others in this group to get involved in issues that affect the group.” The second employee voice scale from Farh et al. (2007) consisted of two items: “This employee actively raises suggestions to improve work procedures or processes” and “This employee actively brings forward suggestions that may help the organization run more efficiently or effectively.”

#### Data Analysis

To again validate the factor structure of the promotive and prohibitive voice scales, we conducted CFAs using AMOS 25.0 (Arbuckle, 2017). Next, we examined the convergent and discriminant validity by means of correlations. We tested measurement invariance across gender by means of multigroup CFA. For all these analyses, we used the sample from Time 1 (*N* = 209). To test the measurement invariance across time, we conducted multigroup CFA with the sample that included data from both Time 1 and Time 2 (*N* = 107).

### Results

#### Factor Structure

Results of the CFAs replicated the results and factor structure found in Study 1: the two-factor model showed an acceptable fit and was significantly superior [Δχ^2^(1) = 251.52, *p* < 0.001] to a one-factor model (see Table 1). In addition, all standardized factor loadings were significant (see Table 2). AVE values for promotive voice (0.64) and prohibitive voice (0.57) were acceptable and provide evidence for convergent validity (Fornell and Larcker, 1981).

#### Descriptive Statistics, Convergent and Discriminant Validity

Table 3 presents the means, standard deviations, intercorrelations, and reliabilities of the variables in this study. Skewness and kurtosis values for the promotive voice items ranged from (−0.93 to −0.69) and (−0.22 to 1.30), respectively. For the prohibitive voice items, skewness and kurtosis values ranged from (−0.80 to −0.39) and (−0.66 to 0.19), respectively. To further test convergent validity, we examined the Pearson correlations between the promotive and prohibitive voice scales and the two alternative employee voice scales. Results showed that both promotive voice and prohibitive voice were positively correlated with LePine and Van Dyne (1998) employee voice scale (*r* = 0.74, *p* < 0.001 and *r* = 0.62, *p* < 0.001, respectively) and with Farh et al.’s (2007) employee voice scale (*r* = 0.67, *p* < 0.001 and *r* = 0.51, *p* < 0.001, respectively), suggesting good convergent validity. In addition, the difference in strength of the correlations was significant for both LePine and Van Dyne (1998) employee voice scale [*r* = 0.74 vs. *r* = 0.62, *t*(206) = −2.81, *p* = 0.005] and Farh et al.’s (2007) employee voice scale [*r* = 0.67 vs. *r* = 0.51, *t*(206) = 3.21, *p* = 0.001]. Together, these results provide support for the convergent validity of the Dutch promotive and prohibitive voice scale.

**TABLE 3:** Descriptives and intercorrelations of the study variables (*N*_*time1*_ = 209).

Variable		Mean	*SD*	1	2	3	4	5	6	7
(1)	Gender	0.64	–							
(2)	Promotive voice	3.80	0.79	−0.23**	*(0.90)*					
(3)	Prohibitive voice	3.65	0.82	−0.24**	0.55**	*(0.86)*				
(4)	Employee voice (LePine and Van Dyne, 1998)	3.70	0.85	−0.17*	0.74**	0.62**	*(0.89)*			
(5)	Employee voice (Farh et al., 2007)	3.84	0.94	−0.12	0.67**	0.51**	0.81**	*(0.89)*		
(6)	Knowledge sharing	4.30	0.60	0.10	0.27**	0.22**	0.40**	0.36**	*(0.80)*	
(7)	Employee silence	1.64	0.60	0.03	−0.32**	−0.48**	−0.43**	−0.46**	−0.41**	*(0.83)*

*Reliability coefficients are on the diagonal.*

*p < 0.01

**p < 0.001.

To test discriminant validity, we examined the Pearson correlations between, on the one hand, promotive and prohibitive voice, and, on the other hand, two theoretically distinct constructs, namely knowledge sharing and employee silence. Table 3 shows that both promotive voice and prohibitive voice show weak positive correlations with knowledge sharing (*r* = 0.27, *p* < 0.001 and *r* = 0.22, *p* = 0.001, respectively) and weak to medium negative correlations with employee silence (*r* = −0.32, *p* < 0.001 and *r* = −0.48, *p* < 0.001, respectively). Together, these findings provide evidence for discriminant validity.

#### Measurement Invariance

We performed a multigroup CFA in several steps to test whether the two-factor structure of the promotive and prohibitive voice scales was invariant across gender and time. Following Vandenberg and Lance (2000), we first created a configural model (M1), by constraining one-factor loading for each subscale and one item intercept to be equal. We then created two nested models where factor loadings (to test for metric invariance; M2), and item intercepts (to test for scalar invariance; M3) were sequentially constrained between groups. Fit indices of the nested models were assessed to probe for invariance. Invariance was assessed using the |ΔCFI| < 0.01 (Cheung and Rensvold, 2002) and the |ΔRMSEA| < 0.01 criteria (Rutkowski and Svetina, 2014).

The results for measurement invariance tests are presented in Table 4. For gender, the configural invariance model (M1) provided an adequate fit, indicating that the two-factor structure of the promotive and prohibitive voice scales is configurally invariant across gender. The metric invariance model (M2) fit the data well and provided support for measurement invariance, with changes in CFI and RMSEA within acceptable criteria values (ΔCFI = −0.001, ΔRMSEA = 0.003). This indicates that the items are measuring the same construct for men and women. The scalar invariance model (M3) did not provide full support for measurement invariance (ΔCFI = 0.021, ΔRMSEA = 0.014). However, after sequentially releasing three item intercepts (backward method) (Yoon and Kim, 2014; Putnick and Bornstein, 2016), the model reached partial scalar invariance (M3a; ΔCFI = 0.007, ΔRMSEA = 0.001).

**TABLE 4:** Reporting tests for measurement invariance.

Model	χ^2^ (df)	CFI	TLI	RMSEA (90% CI)	SRMR	Model comparison	Δχ^2^ (df)	Δ CFI	Δ TLI	Δ RMSEA	Decision
**Gender^a^**											
M1: Configural invariance	128.37** (69)	0.948	0.932	0.064 (0.047–0.082)	0.079						
M2: Metric invariance	144.77** (76)	0.939	0.928	0.066 (0.050–0.082)	0.110	M1	16.40* (7)	0.009	0.004	0.002	Accept
M3: Scalar invariance	178.73** (86)	0.918	0.915	0.072 (0.057–0.087)	0.110	M2	33.96** (10)	0.021	0.014	0.006	Reject
M3a: Partial scalar invariance	160.62** (83)	0.932	0.926	0.067 (0.051–0.083)	0.108	M2	15.86* (7)	0.007	0.002	0.001	Accept
**Time^b^**											
M1: Configural invariance	92.25* (68)	0.980	0.973	0.041 (0.015–0.061)	0.0637						
M2: Metric invariance	98.94* (76)	0.981	0.977	0.038 (0.009–0.057)	0.0622	M1	6.69 (8)	−0.001	−0.004	0.003	Accept
M3: Scalar invariance	104.06 (86)	0.985	0.984	0.031 (0.000–0.051)	0.0623	M2	5.13 (10)	−0.004	−0.007	0.007	Accept

*^a^N_time1_ = 209*

*^b^N_time2_ = 107.*

*p < 0.05

**p < 0.001.

For time, the configural invariance model (M1) fit the data well (see Table 4), indicating that the two-factor structure of the promotive and prohibitive voice scales is configurally invariant across time. The metric invariance model (M2) also fit the data well, with changes in CFI and RMSEA within acceptable values (ΔCFI = −0.001, ΔRMSEA = 0.003). This indicates that all items have the same meaning at different time points. The scalar invariance model (M3) also fit the data well and yielded strong invariance (ΔCFI = −0.004, ΔRMSEA = 0.007), indicating that both factor loadings and item intercepts are invariant across different points in time.

The results of Study 2 (1) provided evidence for the validity of the two-factor structure of the Dutch version of the promotive and prohibitive voice scales (2) provided evidence for its convergent and discriminant validity, and (3) demonstrated measurement invariance partial scalar invariance across gender (i.e., partial scalar invariance) and across time (i.e., scalar invariance).

## Study 3

In Study 3, we further establish the construct validity of the promotive and prohibitive voice scale with other constructs in the nomological network by investigating the relationships with personal initiative, approach temperament, and risk propensity.

### Method

#### Sample and Procedure

We tested our expectations regarding the nomological network of the promotive and prohibitive voice scale in a cross-sectional survey using a convenience sample of employees from several Dutch organizations. Data were collected by a trained master student as part of a research project at the University under the first author’s supervision. Participants were invited by email or via professional network contacts (e.g., LinkedIn) to participate in the study. At the start of the online survey, a cover letter explained the nature of the research and assured participating individuals of anonymity and confidentiality. Participants digitally signed an informed consent. The study was approved by the University’s Ethics Review Board (2017-WOP-7741).

A total of 149 participants completed the questionnaire on all relevant study variables. The majority was female (96 participants; 64,4%). Age ranged from 18 to 63 years, with a mean age of 33.1 years (*SD* = 13.81). Mean tenure was 14.7 years (*SD* = 11.9), with the average tenure in the current organization 5.9 years (*SD* = 6.97).

#### Measures

Unless indicated otherwise, all measures were assessed with 5-point Likert scales ranging from 1 (*strongly disagree*) to 5 (*strongly agree*).

##### Promotive and Prohibitive Voice

Promotive and prohibitive voice were assessed with the 10-item Dutch version of the voice scale as described in Study 1.

##### Approach Temperament

To measure approach temperament, we used the six-item Dutch version of the approach temperament questionnaire (Bipp et al., 2017). Example items are: “When I see an opportunity for something I like, I immediately get excited” and “When I want something, I feel a strong desire to go after it.”

##### Personal Initiative

We measured personal initiative with a 7-item scale (Frese et al., 1997). Example items are: “I am always looking for better ways to do things” and “I excel at identifying opportunities.”

##### Risk Propensity

To assess risk propensity we used a 7-item scale developed by Meertens and Lion (2008). Example items are: “I prefer to avoid risks (*reverse scored*)” and “I usually view risks as a challenge.”

### Results

#### Factor Structure

First, and similar to Study 1 and Study 2, we conducted CFAs using AMOS 25.0 to examine the two-factor structure of the promotive and prohibitive voice scales. Table 1 shows the goodness of fit statistics: the two-factor model provided a good fit and was superior to a one-factor solution [Δχ^2^(1) = 107.64, *p* < 0.001]. The estimated factor loadings are presented in Table 2. AVE values for promotive voice (0.62) and prohibitive voice (0.49). CR values were 0.89 and 0.83 for promotive and prohibitive voice, respectively. Although for prohibitive voice the AVE value is just below the 0.50 cutoff, the CR value is well above 0.70, meaning that convergent validity can still be regarded as adequate (Fornell and Larcker, 1981).

#### Nomological Network

We assessed the nomological validity of the promotive and prohibitive voice scales by examining their (dispositional) antecedents. Table 5 shows the descriptive statistics, intercorrelations and reliabilities of the variables. Skewness and kurtosis values for the promotive voice items ranged from (−0.69 to −0.27) and (−0.62 to 0.13), respectively. For the prohibitive voice items, skewness and kurtosis values ranged from (−0.59 to −0.17) and (−0.76 to −0.12), respectively.

**TABLE 5:** Means, standard deviations, and correlations (Study 3).

	Variable	Mean	*SD*	*1*	*2*	*3*	*4*	*5*	*6*	*7*
(1)	Age	33.13	13.81							
(2)	Gender	0.64	–	0.11						
(3)	Personal initiative	3.77	0.68	0.13	0.01	*(0.87)*				
(4)	Approach temperament	3.80	0.69	−0.01	0.08	0.64**	*(0.81)*			
(5)	Risk propensity	2.84	0.65	−0.02	−0.21*	0.13	0.10	*(0.67)*		
(6)	Promotive voice	3.54	0.87	0.12	−0.01	0.56**	0.53**	0.13	*(0.89)*	
(7)	Prohibitive voice	3.39	0.83	0.10	−0.08	0.49**	0.36**	0.24**	0.55**	*(0.83)*

*N = 149. Reliability coefficients are on the diagonal.*

* p < 0.05

** p < 0.01.

Table 6 presents the results of multiple hierarchical regression analyses with promotive and prohibitive voice as the dependent variable, respectively. In the analysis with promotive voice as the dependent variable, we included prohibitive voice as a covariate and vice versa (Model 1). Personal initiative, approach temperament, and risk propensity were entered in Model 2. In line with our expectations, personal initiative was positively related to both promotive voice (β = 0.23, *p* = 0.007) and to prohibitive voice (β = 0.26, *p* = 0.005). Approach temperament was positively related to promotive voice (β = 0.25, *p* = 0.002) but not to prohibitive voice (β = −0.03, *p* = 0.747). Risk propensity was unrelated to promotive voice (β = −0.02, *p* = 0.834) but positively related to prohibitive voice (β = 0.16, *p* = 0.019). Together, these results provide further support for the construct validity of the Dutch version of the promotive and prohibitive voice scale.

**TABLE 6:** Results of regression analyses.

Variables	Promotive voice		Prohibitive voice	
	Model 1	Model 2	Model 1	Model 2
Promotive voice			0.55**	0.40**
Prohibitive voice	0.55**	0.35**		
Personal initiative		0.23*		0.26**
Approach temperament		0.25*		−0.03
Risk propensity		−0.01		0.16*
Δ*R*^2^	0.30**	0.15**	0.30**	0.07*
Adjusted *R*^2^	0.30**	0.44**	0.30**	0.36**

*N = 149. Standardized regression coefficients are reported for the respective regression steps.*

*p < 0.05

**p < 0.001.

## Additional Analyses

To further support the validity of the two-factor structure of the promotive and prohibitive voice scale, we conducted CFAs using AMOS 25.0 on the combined samples of Study 1, Study 2 (time 1), and Study 3. The goodness of fit statistics are presented in Table 1 and show that the two-factor model provided a good fit and was superior to a one-factor solution [Δχ^2^(1) = 411.72, *p* < 0.001]. The estimated factor loadings are presented in Table 2.

## General Discussion

The primary purpose of this study was to validate the Dutch version of the promotive and prohibitive voice scale and to further establish the psychometric properties and construct validity of the promotive and prohibitive voice scale in general. Results of three separate studies provided unequivocal evidence for the validity and reliability of the Dutch version of the promotive and prohibitive voice scale. In doing so, we replicated findings from the original promotive and prohibitive voice scale (Liang et al., 2012) and further established its construct validity by investigating discriminant validity and advancing the nomological network. Below we will discuss the implications in more detail.

### Theoretical Implications

Across three samples we provided evidence for the two-factorial structure of the promotive and prohibitive voice scale in a Dutch context. The promotive voice dimension and the prohibitive voice dimension showed an excellent internal consistency reliability, i.e., well above the suggested threshold of 0.70. We also demonstrated the convergent validity of the Dutch promotive and prohibitive voice scale: promotive and prohibitive voice were strongly correlated with two alternative employee voice measures, and relatively weakly correlated with theoretically distinct constructs such as knowledge sharing and employee silence. Taken together, these results replicate and extend the reported validity and reliability of the original English version of the scale (cf. Liang et al., 2012).

To further establish the validity of the Dutch version of the promotive and prohibitive voice scale, we also examined its measurement invariance across gender and time. For time, we found full scalar invariance: The items in the promotive and prohibitive voice scale were interpreted similarly at different time points and participants responded in a similar fashion to the items. Put differently, none of the participants responded systematically higher or lower to the items of the promotive and prohibitive voice scale across time (Meredith, 1993; Vandenberg and Lance, 2000). The measurement invariance across time indicates that the promotive and prohibitive voice scale allows for meaningful comparisons between participants over time, which is particularly important for longitudinal research. That is, latent mean differences between different time points can be interpreted as actual time differences. For gender, we found partial scalar invariance: the intercepts of seven out of ten items were equal across gender. Although standards for partial invariance vary, releasing three out of ten item intercepts can be regarded as acceptable (for a discussion see Putnick and Bornstein, 2016). We can therefore conclude that men and women interpreted the items of the promotive and prohibitive voice scale similarly and responded in a similar fashion to the items. Thus, the measurement invariance across gender allows us to interpret latent mean differences between males and females as actual gender differences.

Finally, we tested and extended the divergent nomological network of the promotive and prohibitive voice scale. Our results showed that two dispositional antecedents were differentially related to promotive and prohibitive voice. Specifically, approach temperament was only positively related to promotive voice, while risk propensity was only positively related to prohibitive voice. These findings show that the promotive and prohibitive dimensions of voice have linkages with unique antecedents, underscoring the importance and added value of distinguishing between these two different manifestations of the construct employee voice. Yet, by uncovering these unique associations with promotive and prohibitive voice, our study also signals that previous voice research that did not explicitly account for this distinction may have drawn incomplete conclusions and, as such, is hard to interpret (see also Chamberlin et al., 2017). By extension, we believe that our finding that personal initiative was positively related to both types of voice is relevant as well: while the meta-analytic study by Chamberlin et al. (2017) was unable to test for the differential effects of personal initiative because the included studies did not use a measure that could distinguish between promotive and prohibitive aspects, our study -in which both manifestations of voice could be distinguished- showed that the dispositional characteristic personal initiative was positively related to both promotive and prohibitive voice. This finding implies that voice is a form of proactive behavior, regardless of whether it is suggestion-focused (promotive) or problem-focused (prohibitive). Taken together, our findings show that promotive and prohibitive voice share certain antecedents (e.g., personal initiative) but, at the same time, also have their unique antecedents. As such, our study has paved the road to further define the nomological network for promotive and prohibitive voice and may help to produce a cohesive set of shared and unique antecedents.

### Practical Implications

First and foremost, the results of this study can be useful for researchers that conduct cross-national comparative studies (e.g., between Western countries), cross-cultural studies between the Dutch (Western) culture and another culture (e.g., Eastern-European or Asian), and/or researchers that aim to validate the promotive/prohibitive voice measure in other languages. Second, the validated Dutch version of the promotive/prohibitive voice scale allows Dutch organizations and employees to assess their collective and individual levels of promotive and prohibitive voice, and, by extension, the extent to which there may be room for improvement. Third, our findings indicate that organizations and managers may cultivate and facilitate promotive and prohibitive voice among their employees by encouraging certain characteristics and by creating opportunities and an environment that allows them to engage in voice behavior. For example, organizations can aim to stimulate promotive voice among their employees by encouraging proactivity or stimulating and cultivating approach related motivations. In contrast, focusing on risk propensity within the organization can be relevant if the aim is to stimulate prohibitive voice behavior.

### Limitations and Future Research

Despite the multi-study approach and elaborate assessment of the validity and reliability of the promotive and prohibitive voice scale in this study, some limitations should be taken into account. First, the relatively small sample size in some of the studies did not allow for a test of our hypotheses at a latent measurement level. Second, the cross-sectional nature of Study 3 did not allow to draw causal inferences. Although not directly the purpose of this study, the cross-sectional nature prevented conclusions regarding the directionality of the theoretically suggested relationships. Third, we did not investigate predictive validity of promotive and prohibitive voice in relation to, for example, job performance (Chamberlin et al., 2017). This is unfortunate, given the presumed importance of employee voice for employee performance within organizations. Finally, in this study, we only examined associations between the promotive and prohibitive voice scale and other self-report measures. To further strengthen the convergent validity of the Dutch voice scales, future studies could, for example, examine whether these scales are associated with behavioral measures or supervisor ratings of voice behavior.

## Conclusion

We showed that the Dutch version of the promotive and prohibitive voice scale has excellent psychometric properties in terms of reliability, factor structure, and construct validity (convergent, discriminant, and nomological network). We also found support for strong measurement invariance across gender and time. Thus, this study provides researchers with a validated Dutch version to measure promotive and prohibitive voice and, as such, to advance the field of voice research.

## Data Availability Statement

The raw data supporting the conclusions of this article will be made available by the authors, without undue reservation.

## Ethics Statement

The studies involving human participants were reviewed and approved by Ethics Review Board of the Faculty of Social and Behavioral Sciences of the University of Amsterdam, Netherlands. The patients/participants provided their written informed consent to participate in this study.

## Author Contributions

RS designed the studies, collected the data, and did the data analysis. RS and JK contributed to the writing of the manuscript and approved the submitted version.

## Conflict of Interest

The authors declare that the research was conducted in the absence of any commercial or financial relationships that could be construed as a potential conflict of interest.

## Publisher’s Note

All claims expressed in this article are solely those of the authors and do not necessarily represent those of their affiliated organizations, or those of the publisher, the editors and the reviewers. Any product that may be evaluated in this article, or claim that may be made by its manufacturer, is not guaranteed or endorsed by the publisher.

## Appendix A1

**TABLE A1:** Promotive and prohibitive voice items in Dutch and English.

Items of the Dutch version of the promotive and prohibitive voice scale	Items of the original English version of the promotive and prohibitive voice scale (Liang et al., 2012)
**Instructies voor de respondent:** In hoeverre bent u het eens met de volgende stellingen?	**Instructions for the respondent:** Indicate your agreement with each of the following statement.
**Antwoordopties:** (1) helemaal niet mee eens; (2) enigszins mee oneens; (3) noch oneens, noch eens; (4) enigszins mee eens; (5) helemaal mee eens	**Response options:** (1) strongly disagree; (2) disagree; (3) neither agree nor disagree; (4) agree; (5) strongly agree
**Promotive voice**	**Promotive voice**
(1) Ik ontwikkel en geef op proactieve wijze suggesties voor werk-gerelateerde zaken die mogelijk van invloed zijn op het team.	(1) I proactively develop and make suggestions for issues that may influence the unit.
(2) Ik stel op proactieve wijze nieuwe projecten voor die nuttig zijn voor het team.	(2) I proactively suggest new projects which are beneficial to the work unit.
(3) Ik kom met suggesties om de werkprocessen van het team te verbeteren.	(3) I raise suggestions to improve the unit’s working procedure.
(4) Ik kom met constructieve suggesties die het team helpen bij het bereiken van hun doelen.	(4) I proactively voice out constructive suggestions that help the unit reach its goals.
(5) Ik opper constructieve voorstellen om het functioneren van het team te verbeteren.	(5) I make constructive suggestions to improve the unit’s operation.
**Prohibitive voice**	**Prohibitive voice**
(6) Ik spreek andere collega’s aan op ongewenste gedragingen die de werkprestaties kunnen belemmeren.	(6) I advise other colleagues against undesirable behaviors that would hamper job performance.
(7) Ik spreek me eerlijk uit over problemen die ernstige schade voor het team kunnen veroorzaken, zelfs wanneer hierover afwijkende meningen bestaan.	(7) I speak up honestly with problems that might cause serious loss to the work unit, even when/though dissenting opinions exist.
(8) Ik durf mijn mening te geven over zaken die van invloed kunnen zijn op de efficiëntie binnen het team, zelfs als dit anderen in verlegenheid kan brengen.	(8) I dare to voice out opinions on things that might affect efficiency in the work unit, even if that would embarrass others.
(9) Ik durf te wijzen op problemen wanneer deze verschijnen binnen het team, zelfs als dat de relaties met andere collega’s zou belemmeren.	(9) I dare to point out problems when they appear in the unit, even if that would hamper relationships with other colleagues.
(10) Ik meld proactief coördinatieproblemen op de werkvloer aan het management.	(10) I proactively report coordination problems in the workplace to the management.

## References

[B1] Arbuckle J. L. (2017). *IBM SPSS Amos 25 User’s Guide.* United States: IBM Corporation.

[B2] Argyris C. (1977). Double loop learning in organizations. *Harv. Bus. Rev.* 55 115–125.

[B3] Bipp T. Kleingeld A. Van Dam K. (2017). Approach and avoidance temperament: an examination of its construct and predictive validity at work. *Eur. J. Psychol. Assess.* 33 196–206. 10.1027/1015-5759/a000285

[B4] Brislin R. W. (1986). “The wording and translation of research instruments” in *Field Methods in Cross-cultural Research.* eds Lonner W. J. Berry J. W. (United States: Sage). 137–164.

[B5] Campbell D. T. Fiske D. W. (1959). Convergent and discriminant validation by the multitrait-multimethod matrix. *Psychol. Bull.* 56 81–105. 10.1037/h0046016 13634291

[B6] Chamberlin M. Newton D. W. Lepine J. A. (2017). A meta-analysis of voice and its promotive and prohibitive forms: identification of key associations, distinctions, and future research directions. *Pers. Psychol.* 70 11–71. 10.1111/peps.12185

[B7] Cheng Y. Nudelman G. Otto K. Ma J. (2020). Belief in a just world and employee voice behavior: the mediating roles of perceived efficacy and risk. *J. Psychol. Interdisc. Appl.* 154 129–143. 10.1080/00223980.2019.1670126 31644371

[B8] Cheung G. W. Rensvold R. B. (2002). Evaluating goodness-of-fit indexes for testing measurement invariance. *Struct. Equat. Model.* 9 233–255. 10.1207/S15328007SEM0902_5 33486653

[B9] Cohen J. Cohen P. West S. G. Aiken L. S. (2003). *Applied Multiple Regression/Correlation Analysis for the Behavioral Sciences* 3rd Edn. United States: Lawrence Erlbaum Associates.

[B10] Cronbach L. J. Meehl P. E. (1955). Construct validity in psychological tests. *Psychol. Bull.* 52 281–302. 10.4324/9781315128498 13245896

[B11] Elliot A. J. Thrash T. M. (2010). Approach and avoidance temperament as basic dimensions of personality. *J. Pers.* 78 865–906. 10.1111/j.1467-6494.2010.00636.x 20573129

[B12] Farh J. L. Hackett R. D. Liang J. (2007). Individual-level cultural values as moderators of perceived organizational support-employee outcome relationships in China: comparing the effects of power distance and traditionality. *Acad. Manag. J.* 50 715–729. 10.5465/amj.2007.25530866

[B13] Fornell C. Larcker D. F. (1981). Evaluating structural equation models with unobservable variables and measurement error. *J. Market. Res.* 18 39–50. 10.2307/3151312

[B14] Frese M. Fay D. (2001). Personal initiative: an active performance concept for work in the 21st century. *Res. Organiz. Behav.* 23 133–187. 10.1016/S0191-3085(01)23005-6

[B15] Frese M. Fay D. Hilburger T. Leng K. Tag A. (1997). The concept of personal initiative: operationalization, reliability and validity in two German samples. *J. Occup. Organiz. Psychol.* 70 139–161. 10.1111/j.2044-8325.1997.tb00639.x

[B16] Hinkin T. R. (1998). A brief tutorial on the development of measures for use in survey questionnaires. *Organiz. Res. Methods* 1 104–121. 10.1177/109442819800100106

[B17] Hu L. T. Bentler P. M. (1999). Cutoff criteria for fit indexes in covariance structure analysis: conventional criteria versus new alternatives. *Struct. Equat. Model.* 6 1–55. 10.1080/10705519909540118

[B18] Huang X. Xu E. Huang L. Liu W. (2018). Nonlinear consequences of promotive and prohibitive voice for managers’ responses: the roles of voice frequency and LMX. *J. Appl. Psychol.* 103 1101–1120. 10.1037/apl0000326 29939035

[B19] Ipe M. (2003). Knowledge sharing in organizations: a conceptual framework. *Hum. Resour. Dev. Rev.* 2 337–359. 10.1177/1534484303257985

[B20] Kakkar H. Tangirala S. Srivastava N. K. Kamdar D. (2016). The dispositional antecedents of promotive and prohibitive voice. *J. Appl. Psychol.* 101 1342–1351. 10.1037/apl0000130 27599091

[B21] LePine J. A. Van Dyne L. (1998). Predicting voice behavior in work groups. *J. Appl. Psychol.* 83 853–868.

[B22] Liang J. Farh C. Farh J. L. (2012). Psychological antecedents of promotive and prohibitive voice: a two-wave examination. *Acad. Manag. J.* 55 71–92. 10.5465/amj.2010.0176

[B23] Liu W. Tangirala S. Lam W. Chen Z. Jia R. T. Huang X. (2015). How and when peers’ positive mood influences employees’ voice. *J. Appl. Psychol.* 100 976–989. 10.1037/a0038066 25365730

[B24] Lu L. Lin X. Leung K. (2012). Goal orientation and innovative performance: the mediating roles of knowledge sharing and perceived autonomy. *J. Appl. Soc. Psychol.* 42 E180–E197. 10.1111/j.1559-1816.2012.01018.x

[B25] Meertens R. M. Lion R. (2008). Measuring an individual’s tendency to take risks: the risk propensity scale. *J. Appl. Soc. Psychol.* 38 1506–1520. 10.1111/j.1559-1816.2008.00357.x

[B26] Meredith W. (1993). Measurement invariance, factor analysis and factorial invariance. *Psychometrika* 58 525–543.

[B27] Morrison E. W. (2011). Employee voice behavior: integration and directions for future research. *Acad. Manag. Ann.* 5 373–412. 10.1080/19416520.2011.574506

[B28] Morrison E. W. (2014). Employee voice and silence. *Annu. Rev. Organiz. Psychol. Organiz. Behav.* 1 173–197. 10.1146/annurev-orgpsych-031413-091328

[B29] Morrison E. W. Phelps C. C. (1999). Taking charge at work: extrarole efforts to initiate workplace change. *Acad. Manag. J.* 42 403–419. 10.2307/257011

[B30] Nerstad C. G. L. Searle R. Černe M. Dysvik A. Škerlavaj M. Scherer R. (2018). Perceived mastery climate, felt trust, and knowledge sharing. *J. Organiz. Behav.* 39 429–447. 10.1002/job.2241

[B31] Putnick D. L. Bornstein M. H. (2016). Measurement invariance conventions and reporting: the state of the art and future directions for psychological research. *Dev. Rev.* 41 71–90. 10.1016/j.dr.2016.06.004.Measurement 27942093 PMC5145197

[B32] Reinholt M. Pedersen T. Foss N. J. (2011). Why a central network position isn’t enough: the role of motivation and ability for knowledge sharing in employee networks. *Acad. Manag. J.* 54 1277–1297. 10.5465/amj.2009.0007

[B33] Rutkowski L. Svetina D. (2014). Assessing the hypothesis of measurement invariance in the context of large-scale international surveys. *Educ. Psychol. Measur.* 74 31–57. 10.1177/0013164413498257

[B34] Sherf E. N. Parke M. R. Isaakyan S. (2021). Distinguishing voice and silence at work: unique relationships with perceived impact, psychological safety, and burnout. *Acad. Manag. J.* 64 114–148. 10.5465/amj.2018.1428

[B35] Sitkin S. B. Weingart L. R. (1995). Determinants of risky decision-making behavior: a test of the mediating role of risk perceptions and propensity. *Acad. Manag. J.* 38 1573–1592.

[B36] Svendsen M. Unterrainer C. Jønsson T. F. (2018). The effect of transformational leadership and job autonomy on promotive and prohibitive voice: a two-wave study. *J. Leadersh. Organiz. Stud.* 25 171–183. 10.1177/1548051817750536

[B37] Tangirala S. Ramanujam R. (2008). Exploring nonlinearity in employee voice: the effects of personal control and organizational identification. *Acad. Manag. J.* 51 1189–1203. 10.5465/AMJ.2008.35732719

[B38] Tavakol M. Dennick R. (2011). Making sense of Cronbach’s alpha. *Int. J. Med. Educ.* 2 53–55. 10.5116/ijme.4dfb.8dfd 28029643 PMC4205511

[B39] Van Dyne L. Ang S. Botero I. C. (2003). Conceptualizing employee silence and employee voice as multidimensional constructs. *J. Manag. Stud.* 40 1359–1392.

[B40] Vandenberg R. J. Lance C. E. (2000). A review and synthesis of the measurement invariance literature: suggestions, practices, and recommendations for organizational research. *Organiz. Res. Methods* 3 4–70.

[B41] Wei X. Zhang Z.-X. Chen X.-P. (2015). I will speak up if my voice is socially desirable: a moderated mediating process of promotive versus prohibitive voice. *J. Appl. Psychol.* 100 1641–1652. 10.1037/a0039046 25844927

[B42] Yoon M. Kim E. S. (2014). A comparison of sequential and nonsequential specification searches in testing factorial invariance. *Behav. Res. Methods* 46 1199–1206. 10.3758/s13428-013-0430-2 24356995

